# Control of Sulfide Production in High Salinity Bakken Shale Oil Reservoirs by Halophilic Bacteria Reducing Nitrate to Nitrite

**DOI:** 10.3389/fmicb.2017.01164

**Published:** 2017-06-21

**Authors:** Biwen A. An, Yin Shen, Gerrit Voordouw

**Affiliations:** Petroleum Microbiology Research Group, Department of Biological Sciences, University of Calgary, CalgaryAlberta, AB, Canada

**Keywords:** souring control, shale oil, Bakken, halophilic, nitrate, nitrite, sulfate-reducing bacteria, nitrate-reducing bacteria

## Abstract

Microbial communities in shale oil fields are still poorly known. We obtained samples of injection, produced and facility waters from a Bakken shale oil field in Saskatchewan, Canada with a resident temperature of 60°C. The injection water had a lower salinity (0.7 Meq of NaCl) than produced or facility waters (0.6–3.6 Meq of NaCl). Salinities of the latter decreased with time, likely due to injection of low salinity water, which had 15–30 mM sulfate. Batch cultures of field samples showed sulfate-reducing and nitrate-reducing bacteria activities at different salinities (0, 0.5, 0.75, 1.0, 1.5, and 2.5 M NaCl). Notably, at high salinity nitrite accumulated, which was not observed at low salinity, indicating potential for nitrate-mediated souring control at high salinity. Continuous culture chemostats were established in media with volatile fatty acids (a mixture of acetate, propionate and butyrate) or lactate as electron donor and nitrate or sulfate as electron acceptor at 0.5 to 2.5 M NaCl. Microbial community analyses of these cultures indicated high proportions of *Halanaerobium, Desulfovermiculus, Halomonas*, and *Marinobacter* in cultures at 2.5 M NaCl, whereas *Desulfovibrio, Geoalkalibacter*, and *Dethiosulfatibacter* were dominant at 0.5 M NaCl. Use of bioreactors to study the effect of nitrate injection on sulfate reduction showed that accumulation of nitrite inhibited SRB activity at 2.5 M but not at 0.5 M NaCl. High proportions of *Halanaerobium* and *Desulfovermiculus* were found at 2.5 M NaCl in the absence of nitrate, whereas high proportions of *Halomonas* and no SRB were found in the presence of nitrate. A diverse microbial community dominated by the SRB *Desulfovibrio* was observed at 0.5 M NaCl both in the presence and absence of nitrate. Our results suggest that nitrate injection can prevent souring provided that the salinity is maintained at a high level. Thus, reinjection of high salinity produced water amended with nitrate maybe be a cost effective method for souring control.

## Introduction

Hydraulic fracturing with subsequent production from horizontal wells is used both in shale gas and in shale oil fields (Caper, 2010; Daly et al., 2016; Shrestha et al., 2017). A key difference is that shale oil production can also require injection of water for continued oil recovery (Laurenzi et al., 2016). Flowback waters obtained from shale gas fields are highly saline (Daly et al., 2016; Khan et al., 2016; Shrestha et al., 2017), as are produced waters obtained from shale oil fields (Strong et al., 2013). However, in the case of shale oil fields the salinity of produced water may change depending on the salinity of the injection water used (Shrestha et al., 2017).

The microbial communities in shale gas fields (Ivanova et al., 2011; Tucker et al., 2015; Daly et al., 2016; Ding et al., 2016) have been well characterized. These include the microorganisms introduced during hydraulic fracturing, as well as those indigenous to the formation (Cluff et al., 2014). Shale gas environments are typically rich in hydrocarbons and are at high pressure and temperature (Cluff et al., 2014; Daly et al., 2016; Liang et al., 2016). Previous studies on waters produced from hydraulic fracturing at different shale operations showed similar microbial community composition (Liang et al., 2016; Mouser et al., 2016). Aerobic freshwater microorganisms from the initial fracturing fluids dominated microbial communities in initial flowback waters. These changed to anaerobic halophilic communities with *Firmicutes (Halanaerobium), Bacteroidetes, Beta-, Gamma-, Delta-* and *Epsilonproteobacteria*, along with methanogenic taxa in subsequent flowback waters (Davis et al., 2012; Struchtemeyer et al., 2012; Murali Mohan et al., 2013; Strong et al., 2013; Cluff et al., 2014; Daly et al., 2016; Liang et al., 2016). These communities are capable of hydrocarbon degradation and fermentation at high salinity, including taxa such as *Marinobacter, Halomonas*, and *Halanaerobium* (Cluff et al., 2014; Daly et al., 2016; Liang et al., 2016). *Halanaerobium* in particularly has been linked to biofilm formation and corrosion by degrading the polysaccharide (guar gum) used in the fracturing process while reducing thiosulfate to sulfide (Daly et al., 2016; Liang et al., 2016). Archaeal taxa, are typically detected in later flow back samples, and many are methylotrophic methanogens such as *Methanohalophilus* and *Methanolobus* (Wuchter et al., 2013; Daly et al., 2016). Overall, the microbial communities in shale gas fields, introduced through well drilling or indigenous, have been studied extensively over the past decade.

Contrary to this wealth of knowledge on the microbial communities in shale gas fields, those in shale oil fields are much less well known. This is surprising given that shale oil production from the Bakken formation alone, which spans parts of Saskatchewan and Manitoba in Canada and of North Dakota in the US, is now a mature industry producing 1.2 million barrels of oil per day by 2015 (Laurenzi et al., 2016). In the Bakken formation 0.5 to 3 million gallons (1.9 to 11.4 million liters) of water are required per well (Wang et al., 2016). The oil from the Bakken formation is light (31° to 45° API) (Yevhen et al., 2011), but increasing concentrations of H_2_S have been observed throughout the production process (Yevhen et al., 2011). Souring of Bakken wells can be attributed to geomechanical, thermochemical and biogenic factors (Yevhen et al., 2011). Biogenic souring in conventional oil fields is due to the reduction of sulfate, thiosulfate or sulfur to sulfide by microorganisms (Youssef et al., 2009; Gieg et al., 2011). Souring in oil reservoirs can lead to corrosion and environmental risks. It is not known whether biogenic souring is due to indigenous species or due to microorganisms introduced with the injection fluids. In addition, continuous injection of water to maintain reservoir pressure is not done in shale gas fields. The effect of this process on shale oil fields is currently unknown. High salinity and temperature can inhibit the presence and activity of microorganisms. However decreasing the salinity and temperature of the reservoir may increase biogenic souring potential (Youssef et al., 2009; Gieg et al., 2011). Nitrate injection is often used to control souring by sulfate-reducing bacteria (SRB) in both low temperature and high temperature fields (Reinsel et al., 1996; Gieg et al., 2011; Fida et al., 2016). During nitrate injection, nitrate-reducing bacteria (NRB) can reduce nitrate to nitrite, which can chemically react with sulfide and which inhibits the dissimilatory sulfite reductase (Dsr) in SRB preventing further formation of sulfide (Reinsel et al., 1996; Hubert et al., 2003; Hubert and Voordouw, 2007; Gieg et al., 2011; Fida et al., 2016). In extreme environments, such as at high temperature, nitrite can accumulate with nitrate injection, thus effectively inhibiting souring (Gieg et al., 2011; Fida et al., 2016). Compared to nitrate-mediated souring control at low salinity conditions, there is a lack of knowledge on its effectiveness at high salinity. In the past 4 years we have collected samples from a saline shale oil field in Saskatchewan and the results of our studies on the potential of the associated microbial communities in controlling souring with nitrate are reported here.

## Materials and methods

### Sample collection

Samples were collected from points indicated in Figure 1 in November 2013, January 2015 and August 2015 in 1 L autoclaved Nalgene bottles filled to the brim to exclude air and samples were shipped on ice. Shipping times for these three sets were 4, 3, and 4 days, respectively. The samples were stored in an anaerobic hood with 90% (v/v) N_2_ and 10% CO_2_ (N_2_-CO_2_) atmosphere upon arrival. Four types of samples were sent: source water (SW), injection water (IW), produced waters (PW) with emulsified oil, free-water knockout water (FW) and treatment water (TW). The aqueous layers of the PW samples were used for analyses and enrichments. Synthetic facility water, 300 mL per L of sample, was added to PW samples that had little water and were difficult to separate. Synthetic facility water contained per L: 146 g NaCl, 2.37 g KCl, 1.82 g MgCl_2_, 6.48 g CaCl_2_ and 0.34 g NaHCO_3_.

**Figure 1:**
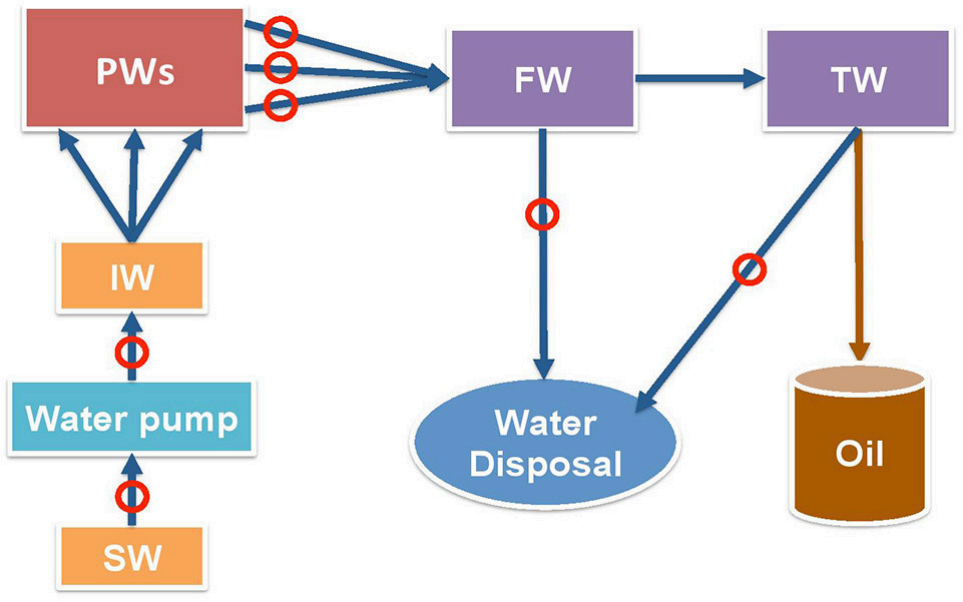
Schematic diagram of shale oil production from a field in the Bakken formation. Mannville formation source water (SW) is injected at the injection well (IW) to pressurize multiple producing wells (PW). Oil-water emulsions from producing wells enter free water knockout (FW) facility first, then treater facility (TW) to separate oil from water. The produced water is not reinjected, but is disposed. Circles indicate the sampling points.

### Analytical determinations and most probable numbers

Water chemistry analyses for field samples were carried out using 50 mL of sample for pH, salinity, sulfate and ammonium concentration measurements. Sulfate concentrations were also measured for enrichments, continuous cultures and bioreactors. Analyses of the concentrations of sulfide, nitrate or nitrite were for enrichments, continuous cultures and bioreactors only. The pH was measured using an Orion pH meter (Model 370). Salinity in molar equivalent (Meq) of NaCl was analyzed with an Orion conductivity cell (model 013005MD). The concentration of dissolved sulfide was measured using the diamine method (Trüper and Schlegel, 1964). Samples were diluted to 1 Meq of NaCl before analyzing sulfate, nitrate and nitrite with the Waters 600E high performance liquid chromatography (HPLC) instrument. Sulfate was measured using a conductivity detector (Waters 423) and IC-PAK anion column (4 × 150 mm, Waters) with acetonitrile, borate/gluconate buffer at a flow rate of 2 ml/min. Nitrate and nitrite were eluted from the same column with the same buffer that were measured with an UV detector (UV/VIS-2489, Waters) at 220 nm. Ammonium concentrations were measured using spectrophotometry with the indophenol method (Aminot et al., 1997).

Viable SRB and acid-producing bacteria (APB) in field samples were enumerated by a miniaturized three well MPN method, using 48 well (6 × 8) plates. For the MPN of SRB, 0.1 ml of sample was inoculated into 0.9 ml of Postgate B medium (at 0.01 M and 2 M NaCl, per L: 0.5 g KH_2_PO_4_, 1.0 g NH_4_Cl, 1.3 g CaSO_4_•2H_2_O, 2.0 g MgSO_4_•7H_2_O, 4.0 g 60% sodium lactate, 1.0 g yeast extract, 0.1 g ascorbic acid, 0.1 g thioglycolate, 0.5 g FeSO_4_•7H_2_O, pH 7-7.5), containing lactate, sulfate and ferrous iron. These were then serially diluted 10-fold to 10^−8^ in the same medium in triplicate wells. The plate was immediately covered with a Titer-Tops membrane and incubated at 32°C inside the anaerobic hood. Wells were scored as positive when a black FeS precipitate was evident. For MPN of APB, the sample was serially diluted in Phenol Red Dextrose medium with 0.1 M or 2 M of NaCl (ZPRA-5, DALYNN Biologicals) using the same procedure as described for SRB. Growth of APB results in a decrease of pH detected as a change in color from orange to yellow. MPN values were calculated for triplicate dilution series by comparing the pattern of positive wells to a probability table for MPN tests (Shen and Voordouw, 2017).

### Media and growth conditions of enrichments and continuous cultures

Enrichments were grown in 120-mL serum bottles with 60 mL of anaerobic Coleville synthetic brine medium K (CSBK) containing the following in g/L: NaCl, 1.5, 29.2, 43.8, 58.4, 87.7, 116.9 or 146.1; KH_2_PO_4_, 0.05; NH_4_Cl, 0.32; CaCl_2_•2H_2_O, 0.21; MgCl_2_•2H_2_O, 0.54; KCl, 0.1. After autoclaving 30 mL of 1 M NaHCO_3_, 1 mL of vitamin solution and 1 mL of selenite-tungstate solution (Hubert et al., 2003) were added, 1 mM of Na_2_S (as reductant) was also added and the pH was adjusted to 7.2 to 7.5 prior to dispensing medium into N_2_-CO_2_ flushed serum bottles. Autoclaved sulfate or nitrate (4 or 10 mM) was added as electron acceptors. Volatile fatty acids (VFA) (3 mM or 6 mM each of acetate, propionate, and butyrate) or lactate (20 mM) was added as the electron donors. Enrichments were incubated at 30°C in the dark shaking at 100 rpm. Enrichments were also used to inoculate continuous cultures (chemostats).

Aliquots (10% v/v) of 2013 and 2015 shale oil field samples were inoculated into 120 mL serum bottles containing 60 mL of reduced CSBK medium with different salinities (0.01, 0.5, 0.75, 1.0, 1.5, 2.0, or 2.5 M NaCl). Incubations received either lactate (20 mM) or VFA (3 or 6 mM) as electron donors and sulfate (10 mM) or nitrate (10 or 20 mM) as electron acceptors. All enrichments were done in duplicate and incubated at 30°C. Successful enrichments were transferred into medium with the same substrates.

Chemostats were inoculated using secondary enrichments of August 2015 field samples (08/15). High salinity (2.5 M NaCl) chemostats contained lactate and sulfate (LS_2.5), lactate and nitrate (LN_2.5), VFA and sulfate (VS_2.5) or VFA and nitrate (VN_2.5). These were inoculated with high salinity enrichments of TW_08/15. Low salinity (0.5 M NaCl) chemostats contained lactate and sulfate (LS_0.5) or lactate and nitrate (LN_0.5). Chemostats contained 100 mL of culture stirring at 400 rpm and were incubated at 37°C. The medium flow rate was generally 42 mL/day, corresponding to a dilution rate of 0.42 day^−1^.

### Bioreactor setup and start-up

**Figure 2:**
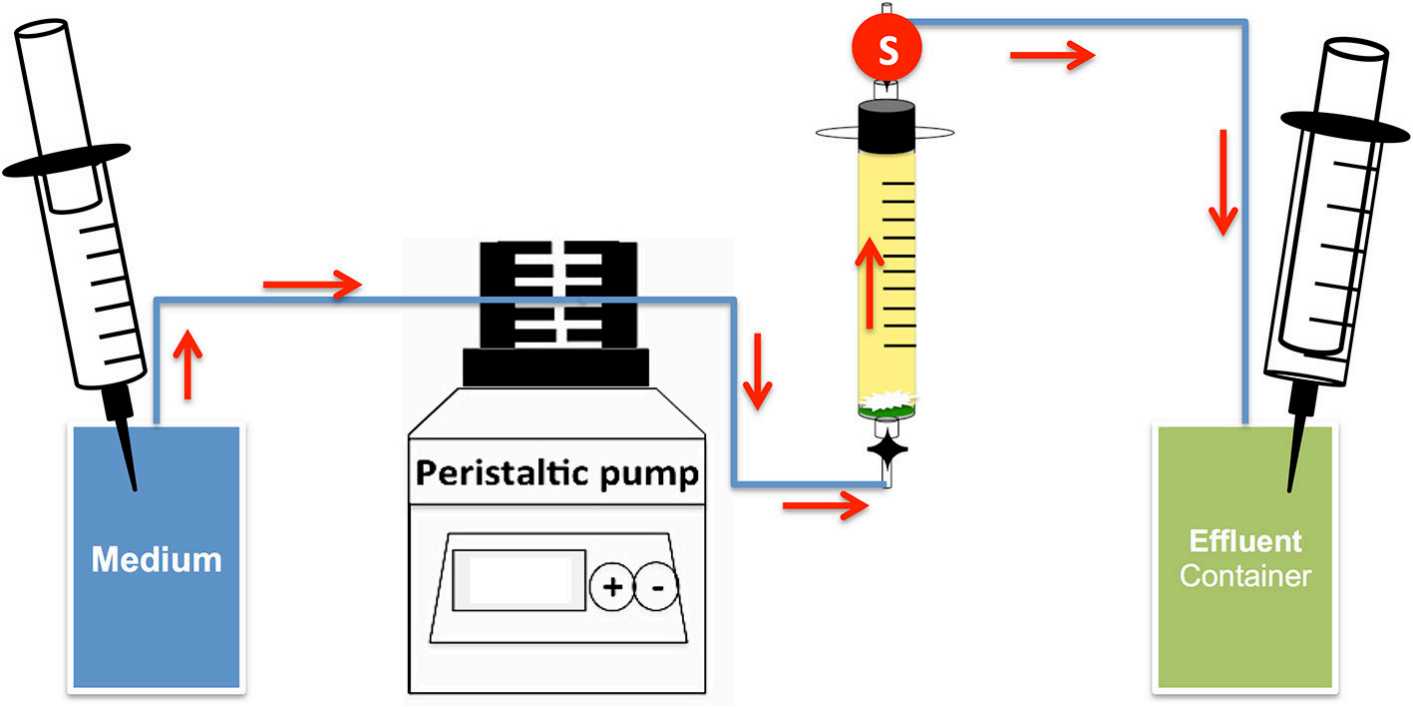
Schematic of up-flow sand packed bioreactor system. The arrows indicate direction of media flow; circle S indicates the effluent sampling point. Pressure in both media and effluent containers was maintained using 30 mL plastic syringes of which the left one was filled with N_2_-CO_2_ gas.

Bioreactors were set up to determine the effect of nitrate on sulfide production under flow conditions at high salt concentration using methods as described previously (Xue and Voordouw, 2015) (Figure 2) with plastic syringes (30 mL) without pistons as bioreactor columns. Glass wool and polymeric mesh were placed at the bottom of the bioreactors. The bioreactors were then tightly packed with sand (Sigma-Aldrich, 50–70 mesh particle size). The pore volume (PV) of the columns was calculated using the weight difference between medium-flooded columns and dry columns (PV = 12.47 ± 0.53 mL). The medium used was CSBK with 0.5 or 2.5 M NaCl and 10 mM sulfate, 10 mM nitrate, 20 mM lactate or 6 mM VFA, as indicated. After media flooding, the bioreactors were inoculated with 0.5 PV of an enrichment or a chemostat culture. Bioreactors were then incubated without flow for 2 weeks. Post incubation, medium with 4 mM sulfate and/or 4 mM nitrate was injected into the bioreactors at flow rates of 0.1 to 0.6 PV/day as indicated. Periodic measurements of anions and sulfide were taken for samples collected from the effluent port of the bioreactors (Figure 2). At the end of the experiment the microbial community compositions were analyzed by Illumina Miseq sequencing. Bioreactors were run at room temperature (22°C).

### DNA extraction and microbial community analyses

DNA was extracted from field samples and for samples from incubations or bioreactors. For field samples, 250 mL of the aqueous phase of each sample was centrifuged at 14,000x g for 20 min at 4°C to pellet the cells. For incubations, 5 mL of sample was centrifuged at 14,000x g for 10 min at 4°C. For the bioreactors, 5 mL of the effluent was collected on ice then centrifuged at 14,000x g for 10 min at 4°C. Following completion of the experiment, the bioreactors were dismantled and 5 mL of sterile water was added to 5 g of inlet sand fraction and shaken vigorously. The supernatant was then removed and centrifuged at 14,000x g for 10 min at 4°C to pellet cells. DNA was extracted from the cell pellets using the FastDNA extraction kit for soil (MP Biomedicals). DNA was quantified with a Qubit fluorimeter (Invitrogen) using the Quant-iT double-stranded DNA (dsDNA) HS assay kit (Invitrogen).

For the 2013 field samples 16S rRNA genes were amplified using a two step-PCR procedure. The first PCR (25 cycles) was done using non-barcoded universal 16S primers (926F: AAACTYAAAKGAATTGRCGG and 1392R: ACGGGCGGTGTGTRC) with conditions as described elsewhere (Park et al., 2011). PCR products were checked with gel electrophoresis and purified with a QIAquick PCR Purification Kit (Qiagen). The second PCR (10 cycles) was done using barcoded FLX titanium amplicon primers 454T_RA_X and 454T_FwB, which have the 16S primers (926F and 1392) as their 3'-ends (Park et al., 2011). The resulting PCR products were purified and quantified prior to pyrosequencing at the McGill University Genome Quebec Innovation Centre, Montreal, using a Genome Sequencer FLX instrument and a GS FLX titanium series XLR70 kit (Roche Diagnostic Corporation).

For 2015 field samples, incubation and bioreactor DNAs were amplified using the same two-step PCR process but with Illumina Miseq non-barcoded primers (926Fi5 TCGTCGGCAGCGTCAGATGTGTATAAGAGACAGAAACTYAAAKGAATWGRCGG and 1392RiF GTCTCGTGGGCTCGGAGATGTGTATAAGAGACAGACGGGCGGTGWGTRC) for the first PCR (25 cycles). For the 2nd PCR (10 cycles), forward primer (P5-S50X-OHAF) with a 29-nt 5' Illumina sequencing adaptor (P5, AATGATACGGCGACCACCGAGATCTACAC), an 8-nt identifying index S50X and a 14-nt forward overhang adaptor (OHAF, TCGTCGGCAGCGTC), and reverse primer (P7-N7XX-OHAF) with a 24-nt 3' Illumina sequencing adaptor (P7, CAAGCAGAAGACGGCATACGAGAT), an 8-nt identifying index N7XX and a 14-nt reverse overhang adaptor (OHAF, GTCTCGTGGGCTCGG), were used. The final PCR product was purified and quantified using the same procedures as above and sent for Illumina Miseq sequencing at the University of Calgary.

Analyses of pyrosequencing and Illumina Miseq sequences were done with the MetaAmp software, (http://ebg.ucalgary.ca/metaamp/; developed by the University of Calgary Energy Bioengineering Group) and sequences were subjected to stringent quality control (QC). Merged reads using PEAR 0.9.8 were uploaded to MetaAmp, which used a cutoff quality score for each sequence of 50 and a minimum length of each sequence of 420 base pairs. The QC sequences were clustered into operational taxonomic units (OTUs) using average neighbor clustering at a distance of 3%. Each remaining OTU was assigned to a taxon by comparing with the latest version of the non-redundant 16S rRNA small subunit SILVA database. Samples were clustered into a dendrogram using the unweighted pair group method algorithm (UPGMA) and the distance between communities was calculated using the Bray-Curtis coefficient in the Mothur software. The dendrogram was visualized using the MEGA5.2.2. Program (Tamura et al., 2011). The entire sets of raw reads have been submitted to the NCBI Sequence Read Archive (SRA) under Bioproject accession number PRJNA181037, with Biosample numbers SAMN06624370 and SAMN06624665.

## Results

### Water chemistry and MPN

The water chemistry of the source water (SW), injection water (IW), produced water (PW), free water knockout water (FW) and treater water (TW) samples is summarized in Table 1. Samples that were mostly oil and required synthetic facility water for obtaining an aqueous extract are not included. SW and IW samples (SW-IWs) had an average salinity of 0.7 ± 0.1 Meq of NaCl, a high sulfate concentration of 29.9 ± 2.6 mM and a low ammonium concentration of 2.0 ± 0.9 mM. These values were similar for all samples reflecting those of the Mannville SW of which the water chemistry was apparently constant with time. In contrast the PWs had salinities ranging from 0.6 to 3.7 Meq of NaCl (average 1.6 ± 1.0 Meq of NaCl), sulfate concentrations from 5.1 to 34.8 mM (average 23.6 ± 10.1 mM) and ammonium concentrations from 2.4 to 33.2 mM (average 17.3 ± 11.0 mM). Three PWs had similar salinity and sulfate concentration as for the SW-IWs. However, only one of these (12PW_08/15) also had a low ammonium concentration of 2.4 mM (Table 1). The bivariate fit analysis of ammonium concentrations and salinity (JMP®, Version *13.1*. SAS Institute Inc., Cary, NC, 1989–2007), with the exception of sample 11PW_08/15, showed a linear relationship (Figure S1: *r*^2^ = 0.90), indicating that the increased salinity and ammonium concentration of the PWs were likely contributed by the shale oil formation. However, salinity and sulfate concentration were not linearly correlated, indicating that the shale formation can either decrease or maintain the sulfate concentration of produced waters from the value of 30 mM (540 ppm) of the SW-IWs (Table 1). The ammonium concentration in flowback waters from shale gas fields was shown to correlate linearly with the chloride concentration in each geological formation with maximum values of 432 ppm (24 mM) of ammonium and 160,000 ppm (4.5 M) of chloride (Harkness et al., 2015). Produced waters from all producing wells are co-mingled in the FW and TW processing facilities. The PWs that were sampled were only a small subset of these. The average salinity of FW and TW (FW-TW) samples was 2.4 ± 0.3 Meq of NaCl. The average ammonium and sulfate concentrations were 22.5 ± 3.5 and 17.2 ± 1.6 mM, respectively. These averages were similar but not identical to those for the PWs, because the selected set of PWs may not represent the average.

**Table 1:** Water chemistry data for the Bakken field samples.

**Sample type**	**Sample name**	**pH**	**Salinity (Meq NaCl)**	**Ion analysis (mM)**	
				**Sulfate**	**Ammonium**
Source waters and injection water	8SW_11/13	6.4	0.7	27.5	1.3
	8SW_01/15	7.2	0.8	33.3	1.3
	8SW_08/15	6.9	0.6	30.2	2.3
	13IW_08/15	7.0	0.8	28.5	3.2
	Average ± Sd	6.9 ± 0.3	0.7 ± 0.1	29.9 ± 2.6	2.0 ± 0.9
Produced waters	5PW_11/13	5.8	2.3	5.1	22.8
	2PW_01/15	6.8	1.2	14.0	5.4
	4PW_01/15	6.5	2.2	15.1	19.0
	2PW_08/15	6.5	0.8	32.5	11.3
	11PW_08/15	6.2	0.6	27.8	33.0
	12PW_08/15	7.3	0.7	30.1	2.4
	14PW_08/15	6.4	3.7	30.3	33.2
	15PW_08/15	6.6	1.1	34.8	11.3
	Average ± Sd	6.5 ± 0.4	1.6 ± 1.0	23.7 ± 10.1	17.3 ± 11.0
Free water knockout and treater water	9FW_11/13	6.0	2.1	17.0	19.2
	10TW_11/13	6.0	2.2	19.2	20.1
	9FW_01/15	6.3	2.8	15.3	19.0
	10TW_01/15	6.3	2.7	17.1	24.3
	9FW_08/15	6.1	2.4	15.8	26.3
	10TW_08/15	6.2	2.3	18.8	26.1
	Average ± Sd	6.1 ± 0.1	2.4 ± 0.3	17.2 ± 1.6	22.5 ± 3.5

*Data for source waters (SW), injection water (IW), produced waters (PW), free-water knockout (FW) and treater water (TW) samples from 2013 (11/13) and 2015 (01/15 or 08/15) are shown*.

MPNs for APB and SRB were determined both at 0.01 M and 2 M NaCl (Table S1). The MPNs were higher at higher salinity for APB in 6 of 7 and for SRB in 4 of 7 PW samples, indicating the presence of a halophilic microbial community.

### Microbial community analysis of field samples

**Figure 3:**
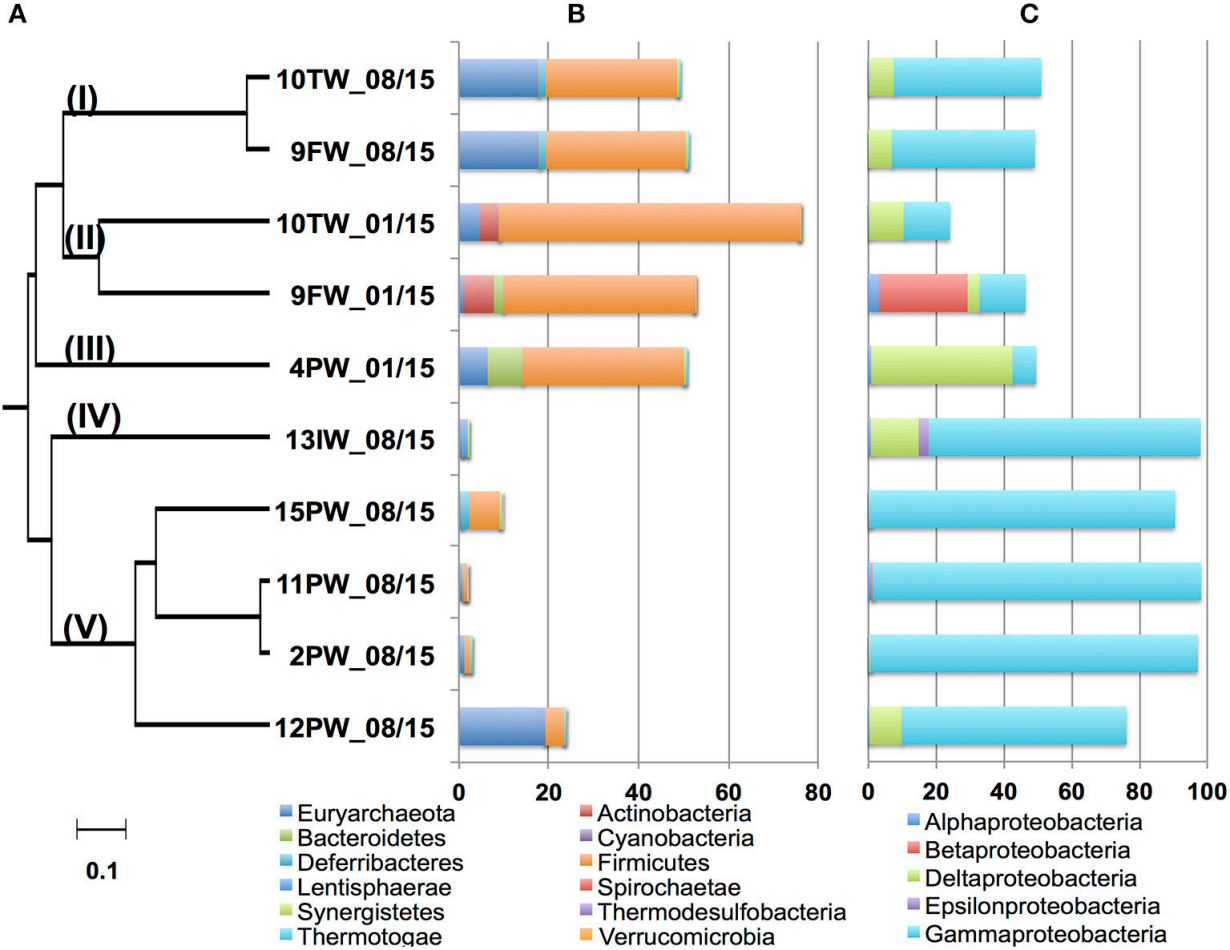
Dendrogram of the 2015 Bakken field samples. The relational tree of microbial community compositions of 2015 samples indicates clades I to V **(A)**. Distribution of phyla other than *Proteobacteria* in the community compositions **(B)**. Distribution of classes from the phylum *Proteobacteria* in the community compositions **(C)**. The scale indicates 10% sequence divergence.

DNAs were isolated, subjected to PCR and either pyrosequencing (2013 samples) or Illumina sequencing (2015 samples). dendrogram for microbial community compositions of the 2015 samples (Figure 3) indicated five distinct clades (Figure 3A; I-V). Communities in FW-TW samples (clades I and II) were distinct from those in most produced water samples (clade V). Microbial community compositions for 4PW and 13IW were in clades III and IV, respectively.

Microorganisms can be described as being halophilic (requiring high salt for growth) or halotolerant (tolerating high salt, but growing better at low salt) (Oren, 2008). Because this property cannot be derived from most genus names, we will use the term halophilic/halotolerant to describe identified taxa. The microbial communities in clade I (FW-TW 08/15) were dominated by the halophilic/halotolerant genera *Halanaerobium, Modicisalibacter, Methanohalophilus*, and *Chromohalobacter*. Microbial communities from clade II (FW-TW 01/15), were dominated by *Halanaerobium, Desulfovermiculus, Methanohalophilus, Rhodococcus, Ralstonia, Marinobacterium*, and *Dethiosulfatibacter* (Table 2). The community compositions of PW samples in clade V had high fractions (45-95%) of *Marinobacter* with smaller fractions of *Halomonas, Thiomicrospira, Methanothermococcus*, and *Desulfotignum* (Table 2). The single IW sample in clade IV had a community dominated by *Thiomicrospira*, unidentified *Gammaproteobacteria* and *Desulfocella* (Figure 3A, Table 2), whereas the single PW sample in clade III had high fractions of *Halanaerobium* (36%) and *Desulfovermiculus* (41%) and smaller fractions of *Methanohalophilus, Chromohalobacter, Marinobacterium*, and *Sphingobacteriales*. Overall these results indicated dominance of halophilic/halotolerant taxa in the microbial communities of all samples, except 13IW.

**Table 2:** Microbial community composition of the Bakken 2015 field samples.

**#Taxonomy (Class; Order; Family; Genus)**	**I**		**II**		**III**	**IV**	**V**			
	**9FW 08/15**	**10TW 08/15**	**9FW 01/15**	**10TW 01/15**	**4PW 01/15**	**13IW 08/15**	**15PW 08/15**	**11PW 08/15**	**2PW 08/15**	**12PW 08/15**
Gammaproteobacteria;Alteromonadales;Alteromonadaceae;Marinobacter;	**2.7**	**1.0**	**8.1**	**1.4**	**1.8**	0.0	**51.0**	**94.5**	**94.6**	**44.6**
Clostridia; Halanaerobiales; Halanaerobiaceae; Halanaerobium;	**31.1**	**29.3**	**31.1**	**66.5**	**36.0**	0.0	**6.6**	0.3	0.8	0.2
Gammaproteobacteria; Thiotrichales; Piscirickettsiaceae; Thiomicrospira;	0.1	0.2	0.0	0.0	0.0	**53.3**	0.0	0.0	0.0	**21.3**
Gammaproteobacteria; Oceanospirillales; Halomonadaceae; Modicisalibacter;	**28.0**	**35.8**	0.0	0.0	0.0	0.0	0.7	0.0	0.0	0.0
Deltaproteobacteria; Desulfovibrionales; Desulfohalobiaceae; Desulfovermiculus;	**1.0**	**1.2**	**1.0**	**6.4**	**40.5**	0.0	0.1	0.0	0.0	0.0
Gammaproteobacteria; Oceanospirillales; Halomonadaceae; Halomonas;	**1.4**	0.9	0.0	0.9	0.0	0.0	**38.2**	**2.1**	**2.0**	0.4
Methanomicrobia; Methanosarcinales; Methanosarcinaceae; Methanohalophilus;	**16.4**	**16.0**	**5.1**	**3.2**	**2.9**	0.0	0.3	0.1	0.1	0.0
Gammaproteobacteria;	0.0	0.0	0.4	0.3	0.0	**26.9**	0.0	0.0	0.0	0.0
Betaproteobacteria; Burkholderiales; Burkholderiaceae; Ralstonia;	0.0	0.0	**25.6**	0.1	0.0	0.0	0.0	0.0	0.0	0.0
Methanococci; Methanococcales; Methanococcaceae; Methanothermococcus;	**1.2**	**1.3**	0.0	**1.4**	**1.4**	0.0	0.1	0.0	**1.0**	**12.7**
Gammaproteobacteria; Oceanospirillales; Halomonadaceae; Chromohalobacter;	**9.8**	**5.5**	0.0	0.0	**1.3**	0.0	0.1	0.0	0.0	0.0
Deltaproteobacteria; Desulfobacterales; Desulfobacteraceae; Desulfocella;	0.0	0.0	0.0	0.1	0.0	**12.0**	0.0	0.0	0.0	0.1
Gammaproteobacteria; Oceanospirillales; Oceanospirillaceae; Marinobacterium;	0.2	0.1	0.0	**9.4**	**2.4**	0.0	0.0	0.0	0.0	0.1
Clostridia; Clostridiales; Clostridiales-Incertae-Sedis; Dethiosulfatibacter;	0.0	0.0	**10.9**	**3.1**	0.0	0.0	0.0	0.0	0.3	0.1
Actinobacteria; Corynebacteriales; Nocardiaceae; Rhodococcus;	0.0	0.0	**4.6**	**4.1**	0.0	0.0	0.0	0.0	0.0	0.0
Deltaproteobacteria; Desulfobacterales; Desulfobacteraceae; Desulfotignum;	0.0	0.0	0.9	0.0	0.0	0.0	0.0	0.0	0.0	**7.7**
Deltaproteobacteria; Desulfovibrionales; Desulfohalobiaceae; Desulfohalobium;	**1.7**	**1.7**	**1.2**	**2.7**	**1.0**	0.0	0.0	0.0	0.2	0.0
Methanobacteria; Methanobacteriales; Methanobacteriaceae; Methanothermobacter;	0.0	0.0	0.9	0.0	**5.2**	0.0	0.0	0.0	0.0	**6.3**
Deltaproteobacteria; Desulfovibrionales;	**3.2**	**3.8**	0.0	0.0	0.0	0.0	0.0	0.0	0.0	0.0
Sphingobacteriia; Sphingobacteriales; E6aC02;	0.0	0.0	0.0	0.0	**6.7**	0.0	0.0	0.0	0.0	0.0
Deferribacteres; Deferribacterales; Deferribacteraceae; Flexistipes;	**1.4**	**1.5**	0.9	0.0	0.0	0.0	**1.8**	0.0	0.1	0.1
Deltaproteobacteria; Desulfovibrionales; Desulfovibrionaceae; Desulfovibrio;	0.3	0.3	0.9	0.9	0.0	**1.2**	0.0	0.0	0.1	**1.6**

*The fraction (%) of Illumina Miseq sequencing reads representing the indicated taxa for samples in 5 distinctive clades (I–V) is shown. Fractions in excess of 1% are indicated in bold*.

The microbial communities in 2013 samples were also dominated by halophilic/halotolerant taxa. High fractions of *Methanohalophilus, Halanaerobium*, and *Desulfovermiculus*, as well as of *Flexistipes, Halomonas*, and *Desulfohalobium* were found (Table S2).

### Reduction of sulfate and nitrate in enrichment cultures at different salinity

**Figure 4:**
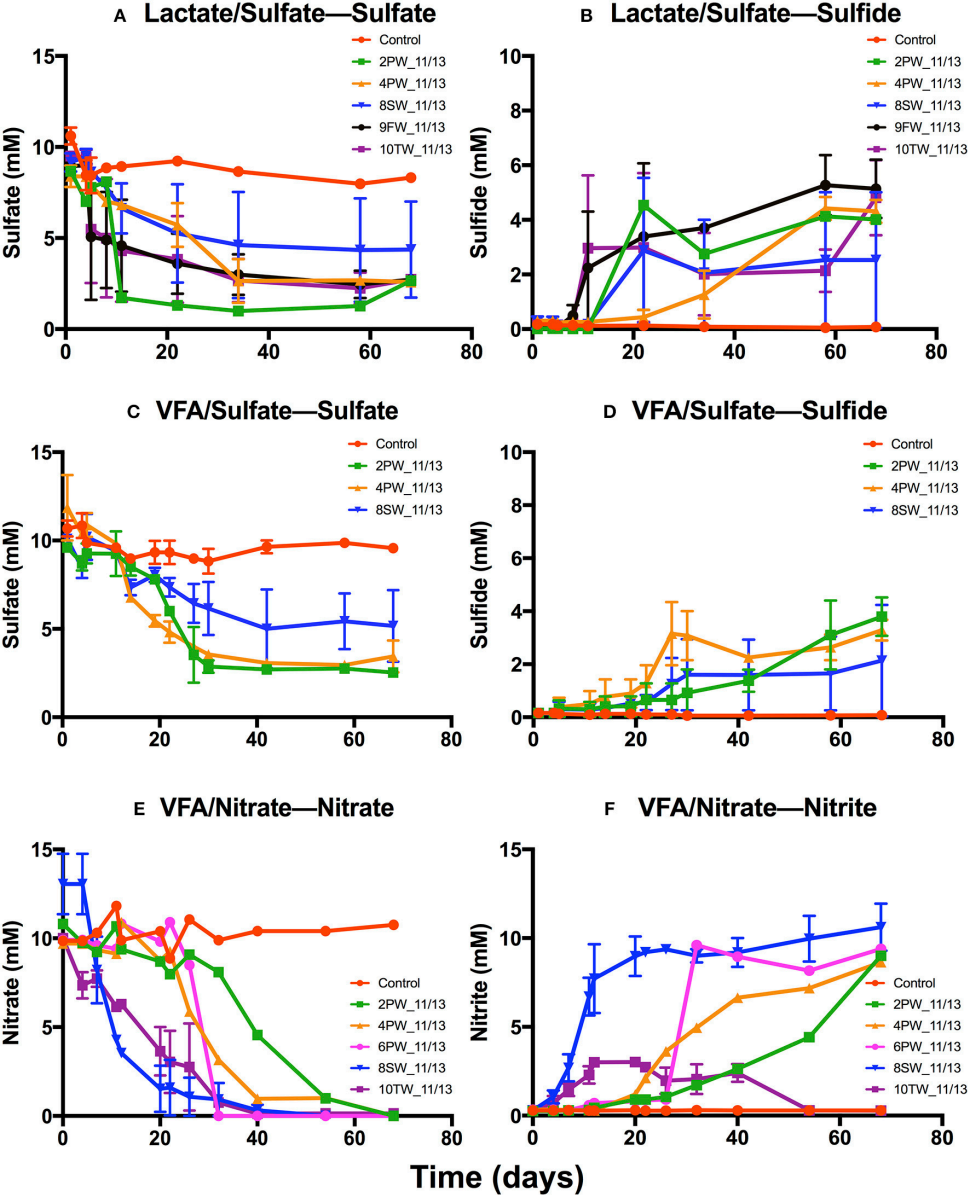
Primary enrichments for November 2013 field samples in media with 2.5 M NaCl. Media contained 10 mM sulfate and 20 mM lactate **(A,B)**, 10 mM sulfate and 3 mM VFA **(C,D)** or 10 mM nitrate and 3 mM VFA **(E,F)**. Media were inoculated with 2PW_11/13, 4PW_11/13, 6PW_11/13, 8SW_11/13, 9FW_11/13, or 10TW_11/13, as indicated; the controls were not inoculated. The average concentrations of sulfide **(B,D)**, sulfate **(A,C)**, nitrate **(E)**, and nitrite **(F)** are shown as a function of time ± SD.

Media with 2.5 M NaCl, containing lactate and sulfate (LS_2.5), VFA and sulfate (VS_2.5) or VFA and nitrate (VN_2.5) were inoculated with 9 field samples collected in 2013. Incubations in LS_2.5 medium showed SRB activity for 5 of these with reduction of 9.5±0.03 mM sulfate to 5.2 ± 0.7 mM of aqueous sulfide in 20 to 60 days (Figures 4A,B), whereas incubations in VS_2.5 medium showed activity for 3 samples with reduction of 9.6 ± 0.2 mM sulfate to 3.8 ± 0.6 mM sulfide (Figures 4C,D). Five samples showed nitrate reduction (Figure 4E). Of these 4 accumulated up to 9.2 ± 0.6 mM nitrite (Figure 4C), whereas nitrite was transiently formed in 10TW_11/13 (Figure 4F).

The enrichments in VS_2.5 medium were used to inoculate fresh cultures at 1.0 M or 2.5 M NaCl with or without 10 mM nitrate. With 4PW_11/13 enrichment sulfide was produced at 1.0 or 2.5 M NaCl, irrespective of the presence of nitrate (Figure S2B). Nitrate reduction was not observed (Figures S2C,D). Similar results were observed for 9FW_11/13 enrichment (Figure S3). In incubations with 10TW_11/13 enrichment at 2.5 M NaCl sulfate was reduced to sulfide in the absence, but not in the presence of nitrate (Figures S4A,B). At 1 M NaCl nitrate was reduced first with little remaining at 15 days and little nitrite being formed (Figures S4C,D). Following the reduction of nitrate, sulfate was reduced with formation of 6.6 ± 0.01 mM sulfide (Figures S4A,B). These results suggest accumulation of nitrite at high salinity (2.5 M NaCl), which inhibits sulfate reduction. At low salinity no nitrite accumulated and no inhibition of sulfate reduction was observed. The results with enrichments of 4PW_11/13 (Figure S2) and 9FW_11/13 (Figure S3) indicated that NRB were absent. These were also absent from the primary enrichment of 9FW_11/13 in VN_2.5 medium (not shown). The primary enrichment of 4PW_11/13 did have NRB activity (Figure 4F).

Only 3 out of 6 samples collected in January 2015 showed SRB activity in LS_2.5 medium with 10.0 ± 0.2 mM sulfide being produced within 60 days (Figure S5). Both LS_0.75 and LS_2.5 were inoculated with 9 field samples collected in August 2015. SRB activity was observed in 6 of 9 samples with production of up to 9.3 and 10.7 mM sulfide at 0.75 and 2.5 M NaCl, respectively (Figures S6A,B). To test the effect of salinity on NRB activity, samples 2PW_08/15, 10TW_08/15, 13W_08/15, and 15PW_08/15 were inoculated into media with VFA and nitrate with 0, 0.5, 1.5 or 2.5 M NaCl (Figure S7). Nitrate reduction was slowest at 2.5 M NaCl with nitrite accumulating in incubations with 2PW_08/15, 10TW_08/15, and 15PW_08/15 (Figures S7A,B,D). Little nitrate reduction and no nitrite accumulation were observed at 2.5 M NaCl in the incubation of 13IW_08/15 (Figure S7C), indicating this sample to have few halophilic NRB. Nitrate reduction was faster at 1.5 M NaCl; nitrite accumulated only in the incubation with 10TW_08/15. Nitrate reduction was fastest at 0.5 and 0 M NaCl, with no nitrite accumulation in any of the incubations (Figure S7).

Thus, the results showed that samples from Bakken shale oil fields harbored halophilic SRB and NRB, capable of growth at 2.5 M NaCl. Halophilic NRB reduced nitrate mostly to nitrite under these conditions. At lower salinities nitrite accumulation was not observed, indicating reduction of nitrate to N_2_. Production of N_2_O was tested by headspace gas analyses, but was not found (results not shown).

### Reduction of sulfate and nitrate in continuous cultures at different salinity

Chemostats at salinities of 0.5 and 2.5 M NaCl were established using secondary enrichments of 13IW_08/15 and 10TW_08/15, respectively. Steady state SRB activity was established within 2 days in LS_0.5-fed, within 50 days in LS_2.5-fed and within 60 days in VS_2.5-fed chemostats (Figures S8A–C). Likewise, steady state NRB activity was established more rapidly at low salinity in LN_0.5 medium (Figure S8F) than at high salinity in LN_2.5 and VN_2.5 media (Figures S8D,E). The average nitrite concentrations in these chemostats were 0.3±0.6, 2.0 ± 2.4 and 1.4 ± 1.5 mM, respectively. Although nitrate concentrations fluctuated significantly they were on average higher at high than at low salinity (Figures S8D–F).

The microbial community compositions in these six chemostats were very different (Table 3). Under sulfate-reducing conditions SRB of the genus *Desulfovibrio* dominated in LS_0.5, whereas *Halanaerobium* and the SRB *Desulfovermiculus* were most prominent at high salinity in LS_2.5 and VS_2.5 (Table 3). Under nitrate-reducing conditions *Geoalkalibacter, Mollicutes* and *Dethiosulfatibacter* dominated in LN_0.5, whereas *Halomonas, Marinobacter* and *Halanaerobium* dominated in LN_2.5 and VN_2.5 (Table 3).

**Table 3:** Microbial community composition of the chemostat enrichments.

**#Taxonomy (Phylum; Class; Order; Family; Genus)**	**Sulfate-reducing**			**Nitrate-reducing**		
	**0.5 M NaCl LS**	**2.5 M NaCl LS**	**2.5 M NaCl VS**	**0.5 M NaCl LN**	**2.5 M NaCl LN**	**2.5 M NaCl VN**
Firmicutes; Clostridia; Halanaerobiales; Halanaerobiaceae; Halanaerobium;	0.1	**86.0**	**31.1**	0.0	**12.9**	**9.4**
Proteobacteria; Gammaproteobacteria; Oceanospirillales; Halomonadaceae; Halomonas;	0.9	0.2	0.4	0.5	**61.5**	**63.7**
Proteobacteria; Deltaproteobacteria; Desulfovibrionales; Desulfovibrionaceae; Desulfovibrio;	**89.3**	0.1	0.0	0.8	0.0	0.0
Proteobacteria; Deltaproteobacteria; Desulfuromonadales; Geobacteraceae; Geoalkalibacter;	0.0	0.0	0.0	**74.1**	0.3	0.1
Proteobacteria; Deltaproteobacteria; Desulfovibrionales; Desulfohalobiaceae; Desulfovermiculus;	0.0	**11.6**	**64.2**	0.0	0.0	0.0
Proteobacteria; Gammaproteobacteria; Alteromonadales; Alteromonadaceae; Marinobacter;	0.8	0.2	**2.2**	0.9	**24.6**	**26.5**
Tenericutes; Mollicutes; NA NB1-n;	**3.3**	0.0	0.0	**14.0**	0.0	0.0
Firmicutes; Clostridia; Clostridiales; no Clostridiales-Incertae-Sedis; Dethiosulfatibacter;	**1.5**	0.1	0.1	**8.8**	0.0	0.0
Proteobacteria; Epsilonproteobacteria; Campylobacterales; Campylobacteraceae; Arcobacter;	**2.5**	0.0	0.0	0.4	0.5	0.3
Firmicutes; Clostridia; Clostridiales; Peptococcaceae;	**1.1**	0.0	0.0	0.0	0.0	0.0

*The fraction (%) of Illumina Miseq sequencing reads representing the indicated taxa is shown. Fractions in excess of 1% are indicated in bold*.

### Control of SRB-mediated souring in bioreactors

SRB enrichments of 4PW_11/13, which had been transferred three times, were used to inoculate two bioreactors. These were then continuously injected with either VSN_2.5 or VS_2.5, as indicated (Figure S9). Injection of VS_2.5, containing 6 mM VFA, 4 mM sulfate and 2.5 M NaCl gave production of 2 mM sulfide after 120 days (Figure S9B), indicating the potential of souring at high salinity. The bioreactor injected with VSN_2.5, containing 6 mM VFA, 4 mM sulfate, 4 mM nitrate and 2.5 M NaCl gave reduction of 2 mM sulfate at day 75. Nitrate reduction did not start until day 110. No nitrate was observed in the bioreactor effluent from day 150 onwards. However, this did contain 2 mM nitrite. As a result sulfate reduction was no longer observed (Figure S9A). The slow start of nitrate reduction indicated that the halophilic SRB enrichment contained few residual NRB at the beginning of the experiment.

**Figure 5:**
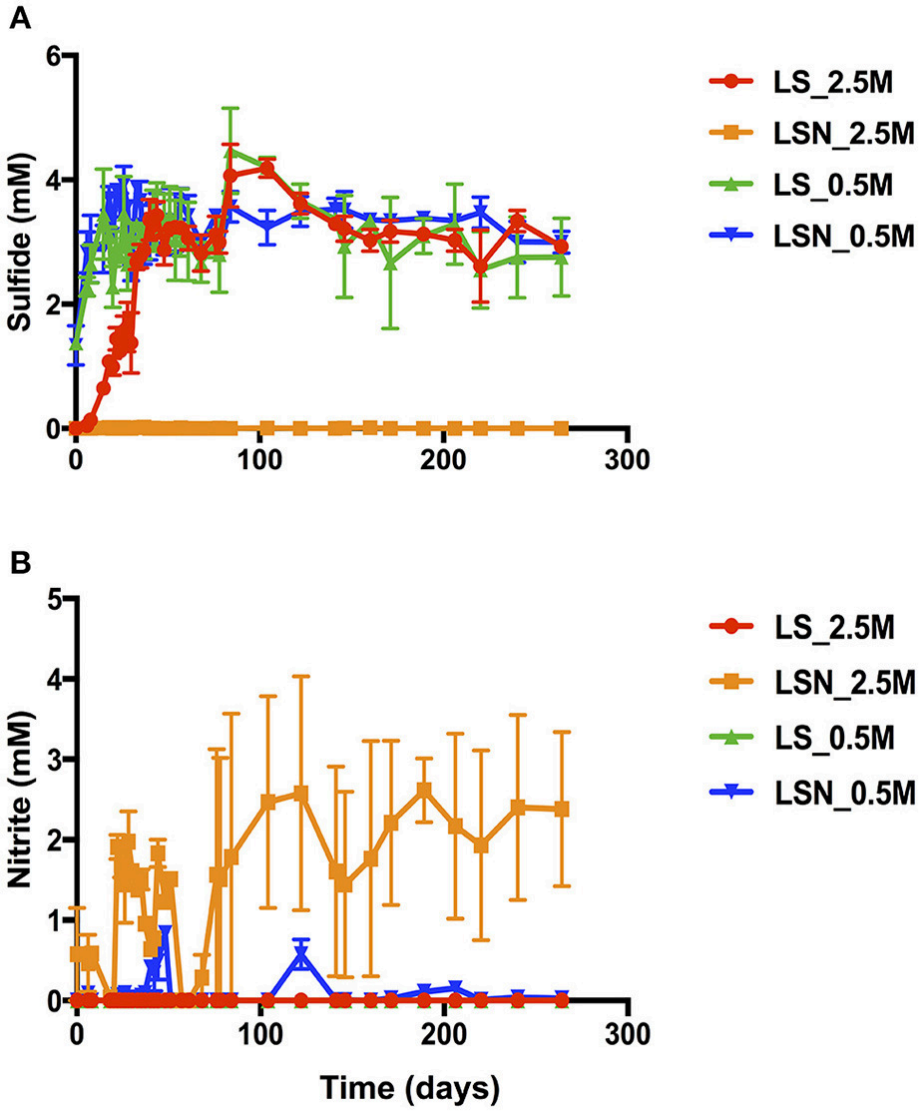
Effect of nitrate on sulfide production in the effluent of bioreactors at low and high salinity. High salinity bioreactors were inoculated with high salinity chemostat cultures and were injected with LSN_2.5 or with LS_2.5. Low salinity bioreactors were inoculated with low salinity chemostat cultures and were injected with LSN_0.5 or with LS_0.5. The concentrations ± SD of sulfide **(A)** and nitrite **(B)** are shown as a function of time. Bioreactors were run at a flow rate of 0.6 PV/day.

Reduction of nitrate and sulfate in bioreactors inoculated with the chemostat cultures of Figure S8 is shown in Figure 5. The duplicated bioreactors (I and II) were continuously injected with LS_0.5, LS_2.5, LSN_0.5, or LSN_2.5, containing 20 mM lactate and 4 mM sulfate without or with 4 mM nitrate. In the absence of nitrate after 30 days, steady concentrations of 3.2 ± 0.4 and 3.2 ± 0.2 mM sulfide were formed at 0.5 and 2.5 M NaCl, respectively (Figure 5A). In the presence of 4 mM sulfate and 4 mM nitrate at low salinity no nitrate or nitrite were detected in the effluent, and steady formation of 3.3 ± 0.2 mM sulfide was observed after 30 days. These results indicated sequential reduction of nitrate and sulfate, as observed elsewhere (Callbeck et al., 2011; Chen et al., 2017). In the presence of 4 mM sulfate and 4 mM nitrate at high salinity no reduction of sulfate to sulfide was detected, whereas all nitrate was reduced. On average 2.4 ± 1.0 mM nitrite persisted in the effluent of LSN_2.5 (Figure 5B). Thus, as in experiments with batch cultures and continuous cultures, nitrite also accumulated in bioreactors at high salinity preventing reduction of sulfate to sulfide (Figure 5A).

The microbial community in influent sand of the LS_2.5 bioreactors had high fractions of *Halanaerobium* and *Desulfovermiculus* (Table 4), while that of LSN_2.5 bioreactors had high fractions of *Halomonas* and *Halanaerobium*, but mostly lacked the halophilic SRB *Desulfovermiculus* (Table 4). At 0.5 M NaCl, with and without nitrate the dominant taxa were *Halanaerobium, Halomonas*, and *Desulfovibrio* (Table 4). A high fraction of *Pseudomonas* was also detected in influent sand of the LSN_0.5 bioreactors, but not in that of LS_0.5 bioreactors (Table 4). Overall, the results from these bioreactors suggest that an accumulation of nitrite at high salinity inhibited growth of the SRB *Desulfovermiculus*. At 0.5 M NaCl *Desulfovibrio* was found as a major community component both in the absence and presence of nitrate. In the presence of nitrate this taxon likely grew in a zone, which was already depleted of nitrate, allowing sulfate reduction.

**Table 4:** Microbial community compositions of the 2015 high and low salinity bioreactors.

**#Taxonomy (Class; Order; Family; Genus)**	**High salinity (2.5 M NaCl)**				**Low salinity (0.5 M NaCl)**			
	**LS I**	**LS II**	**LSN I**	**LSN II**	**LS I**	**LS II**	**LSN I**	**LSN II**
Clostridia; Halanaerobiales; Halanaerobiaceae; Halanaerobium;	**83.5**	**74.3**	**13.7**	**23.0**	**30.1**	**14.0**	**34.3**	**41.7**
Gammaproteobacteria; Oceanospirillales; Halomonadaceae; Halomonas;	**1.3**	**7.0**	**83.2**	**74.7**	**6.2**	**12.0**	**9.8**	**19.9**
Deltaproteobacteria; Desulfovibrionales; Desulfovibrionaceae; Desulfovibrio;	0.0	0.0	0.0	0.0	**54.5**	**68.0**	**31.2**	**29.8**
Gammaproteobacteria; Pseudomonadales; Pseudomonadaceae; Pseudomonas;	0.0	0.0	0.0	0.0	0.0	0.1	**18.1**	**6.8**
Deltaproteobacteria; Desulfovibrionales; Desulfohalobiaceae; Desulfovermiculus;	**10.1**	**9.4**	0.3	0.2	0.0	0.0	0.0	0.0
Clostridia; Clostridiales; Clostridiales-Incertae-Sedis; Dethiosulfatibacter;	0.2	0.2	0.0	0.0	**5.6**	**1.8**	**2.7**	0.5
Gammaproteobacteria; Chromatiales; Halothiobacillaceae; Halothiobacillus;	**1.5**	**3.2**	0.7	**1.4**	0.0	0.0	0.0	0.0
Kazan-3B-09;	**2.2**	**3.3**	0.0	0.0	0.0	0.00	0.0	0.0
Tenericutes; Mollicutes; NB1-n;	0.0	0.0	0.0	0.0	**1.6**	**1.5**	**2.4**	0.0
Clostridia; Clostridiales; Peptococcaceae;	0.0	0.0	0.0	0.0	**1.5**	**2.0**	0.8	**1.0**
Gammaproteobacteria; Alteromonadales; Alteromonadaceae; Marinobacter;	0.1	0.0	**2.0**	0.5	0.0	0.0	0.1	0.0
Deltaproteobacteria; Desulfobacterales; Desulfobacteraceae; Desulfosalsimonas;	0.2	**1.5**	0.0	0.0	0.0	0.0	0.0	0.0

*The dendrogram for duplicated (I or II) bioreactors (0.5 M and 2.5 M NaCl) is shown on the top. The fraction (%) of Illumina Miseq sequencing reads representing the indicated taxa for duplicated (I or II) bioreactors (0.5 M and 2.5 M NaCl) is shown. Fractions in excess of 1% are indicated in bold*.

## Discussion

Our results on microbial activities and community compositions with Bakken shale oil field samples suggest a high potential for souring at both low and high salinities. However, high salinity souring can be effectively controlled by nitrate injection because nitrite accumulates. This is similar to the accumulation of nitrite found in samples from oil fields at or above 50°C (Fida et al., 2016). Thus, NRB limit reduction of nitrate to nitrite under extremophilic conditions, either high temperature or high salinity. Control of H_2_S production by limited reduction of nitrate to nitrite at high salinity has not been previously demonstrated.

Assuming souring to be a problem in shale oil fields (Yevhen et al., 2011) and that nitrate injection is promising technology to remedy this problem, decreases in salinity should be avoided. The salinity of produced waters obtained in the present study varied from 0.6 to 3.7 Meq of NaCl. This was likely caused by continuous injection of SW-IW, which had 0.7 Meq of NaCl. Low salinity in produced waters may signal breakthrough of SW-IW, e.g., 12PW_08/15 had similar salinity, sulfate and ammonium concentrations as SW-IW (Table 1). This was also reflected in similarly high factions of *Thiomicrospira*, a moderately halophilic sulfur-oxidizing microorganism (Brinkhoff and Kuever, 1999) in the microbial communities of 13IW_08/15 (53%) and 12PW_08/15 (21%). This taxon was mostly absent from the communities in other samples (Table 2).

Community compositions of SW-IW samples were dominated by microorganisms, which grow optimally at moderate salinities (Table 2, Table S2) such as *Methanolobus* (optimum 0.35 M NaCl; Michimaru et al., 2009), *Thiomicrospira* (optimum 0.47 M NaCl; Brinkhoff and Kuever, 1999), *Desulfovibrio* (optimum 1.0 M NaCl for halophilic *Desulfovibrio*; Tardy-Jacquenod et al., 1998), *Methanobacterium* (optimum 0.1 M NaCl with tolerance of up to 1.7 M NaCl for some strains; Cadillo-Quiroz et al., 2014) and *Pelobacter* (minimally 0.2 M NaCl; Nasaringarao and Häggblom, 2007). Of these members of the genus *Desulfovibrio* are SRB known to be involved in reservoir souring and microbially influenced corrosion (Hubert et al., 2009; Kakooei et al., 2012; Guan et al., 2014) at low salinity and low temperature conditions. The presence of sulfur-reducers such as *Pelobacter* can further increase sulfide concentrations. Overall, the microbial communities in SW-IW samples had high potential to be involved in reservoir souring at low salinities.

Microbial community compositions of PWs were dependent on salinity. In samples from high salinity Bakken PWs, such as 2PW_11/13, 4PW_11/13 and 4PW_01/15 (2.2 Meq of NaCl), obligately anaerobic taxa, which are halophilic or halotolerant were detected, such as *Halanaerobium, Desulfovermiculus*, and *Desulfohalobium* (Table 2, Table S2). This suggests fermentative and souring potential at high salinity. Archaeal taxa such as *Methanohalophilus Methanothermococcus*, and *Methanothermobacter* were also detected in these high salinity PW samples (Table 2), suggesting potential methanogenic activities. However, PW samples with low salinity, such as 2PW_08/15, 11PW_08/15, 12PW_08/15 (0.7 Meq of NaCl), had decreased fractions of *Halanaerobium* and increased fractions of *Marinobacter*, which grows optimally at lower salinities (Guo et al., 2007). Sample 15PW_08/15 with intermediate salinity (1.1 Meq of NaCl) had an intermediate fraction of *Halanaerobium* (Table 2: 6.6%). Hence, the decrease in PW salinities is reflected in the microbial community compositions.

Samples from the FW-TW facility, which processes oil-water emulsions from all (>100) producing wells, had high salinity throughout. These were dominated by the same obligately anaerobic, halophilic taxa as found in high salinity PWs (Table 2, Table S2: *Halanaerobium, Methanohalophilus, Desulfovermiculus*, and *Desulfohalobium*). The dominant SRB *Desulfovermiculus* and *Desulfohalobium* reduce sulfate using oil organics (butyrate and propionate) and H_2_ (Belyakova et al., 2006; Jakobsen et al., 2006). The fermentative hydrogen producer *Halanaerobium* could enhance this sulfate reduction by providing H_2_ and osmotic solutes (Daly et al., 2016). Other halophilic heterotrophic taxa such as *Modicisalibacter, Halomonas, Chromohalobacter*, and *Marinobacter* were found in FW-TW samples in more variable fractions (Table 2). These can grow fermentatively, but can also reduce nitrate (Vreeland et al., 1980; Gam et al., 2007).

Primary enrichments of SRB using 2013 or 2015 field samples produced sulfide at both low and high salinities (Figure 4, Figures S3,S4). The dominant taxa in these enrichments were *Halanaerobium, Desulfovibrio, Desulfovermiculus, Desulfohalobium*, and *Marispirillum* (data not shown). SRB enrichments at both 0.75 and 2.5 M NaCl were very similar in terms of sulfide formation and microbial community composition, suggesting the potential for souring at both SW-IW and FW-TW salinities (Figure S6). Nitrate reduction at high salinity gave nitrite accumulation (Figures 4E,F, Figure S7), which facilitated souring control (Figure S4), since nitrite prevents H_2_S production by SRB (Callbeck et al., 2011; Gieg et al., 2011). At lower salinities (0, 0.5 and 1.0 M NaCl) nitrite did not accumulate (Figures S4,S7) and was likely reduced further to N_2_, indicating that souring control through nitrate injection will be less effective under these conditions. Incomplete nitrate reduction with the accumulation of nitrite has also been observed at high temperature (Reinsel et al., 1996; Fida et al., 2016; 50°C or higher), indicating that this may generally occur at extreme salinity or temperature.

In contrast to the results with primary enrichments, continuous cultures showed distinct microbial community compositions at high and low salinity (Table 3). At high salinity under sulfate-reducing conditions the microbial community was dominated by *Halanaerobium* and *Desulfovermiculus*. The co-occurrence of these two taxa suggests a possible syntrophic relationship with *Desulfovermiculus* using H_2_ produced by *Halanaerobium* for sulfate-reduction (Belyakova et al., 2006). At low salinity *Halanaerobium* and *Desulfovermiculus* were mostly absent in the LS_0.5 chemostat, where the dominant SRB was *Desulfovibrio*. The complete turnover of microbial compositions between high and low salinity reflects what might happen in the reservoir as low salinity SW-IW is continuously injected. The microbial community composition in nitrate-reducing chemostats at high salinity, LN_2.5 and VN_2.5, were nearly identical with *Halanaerobium, Halomonas*, and *Marinobacter* being dominant in both (Table 3). Of these *Halomonas* and *Marinobacter* are capable of reducing nitrate under saline conditions (Vreeland et al., 1980; Rani et al., 2017). At low salinity the continuous culture in LN_0.5 medium had a very different community (Table 3). This was dominated by *Geoalkalibacter*, an anaerobic nitrate-, sulfur- and iron-reducing microorganism, which grows optimally at 0.3 M NaCl (Greene et al., 2009). The distinctive separation of microbial communities in low salinity and high salinity chemostats correlates to field samples. The data also showed that repeated transferring of high salinity SRB enrichments causes a loss in nitrate-reducing taxa.

The bioreactor studies aimed to determine the feasibility of nitrate-mediated souring control at high salinity under flow conditions, as in the field. A problem with these studies was that halophilic NRB activity is lost from bioreactors, which are operated under sulfate-reducing conditions at high salinity for a prolonged period of time. E.g. in the bioreactor in Figure S9A, injected with nitrate and sulfate, NRB activity did not start until after more than 100 days of operation. Continuously injecting low concentrations of nitrate at high salinity led to nitrite accumulation and souring control (Figure 5B, Figure S9). The microbial communities in bioreactors injected with LS_2.5 continuously produced 3 mM sulfide and were dominated by *Halanaerobium* and *Desulfovermiculus*. In bioreactors injected with LS_0.5 a similar H_2_S production was achieved by a different community dominated by *Desulfovibrio*. In the presence of nitrate LSN_2.5 bioreactors showed no sulfide production throughout the experiment, whereas sulfide was observed in the LSN_0.5 bioreactors. This was due to accumulation of nitrite in the LSN_2.5 bioreactors, which was not observed in the LSN_0.5 bioreactors (Figure 5B). *Halomonas*, which dominated the community in the LSN_2.5 injected bioreactors (Table 3: 75–83%), reduces nitrate only to nitrite (Vreeland et al., 1980). Excess concentrations of electron donors (VFA or lactate) were present throughout in these and other bioreactor studies (Callbeck et al., 2011). Thus, lack of sulfate reduction and incomplete reduction of nitrate to nitrite were not caused by a shortage of electron donor. The sulfide concentration in the LSN_0.5 injected bioreactors was comparable to that of the LS_0.5 injected bioreactors. These had similar communities with high fractions of *Desulfovibrio*, indicating the presence of a zone of sulfate reduction in the LSN_0.5 bioreactors with similar SRB as in the LS_0.5 bioreactors.

Overall, our results have demonstrated that souring in Bakken shale oil reservoirs is highly likely, especially with the ongoing injection of low salinity water with a high sulfate concentration. Reinjection of high salinity produced water amended with nitrate can decrease souring, while potentially improving water disposal issues. This is similar to high temperature oil fields, where reinjection of hot produced water is recommended to avoid lowering the reservoir temperatures to values below 50°C, where nitrite is reduced to N_2_ (Fida et al., 2016).

## Author contributions

BA: Experiment setup, data collection, interpretation, execution of the experiments, drafting and revision of the manuscript. YS: Conducted the experiments for Most Probable Numbers (MPN). GV: Provided funding, supervision, conception of the work and final approval of manuscript version to be published.

### Conflict of interest statement

The authors declare that the research was conducted in the absence of any commercial or financial relationships that could be construed as a potential conflict of interest.
