# Supplementary material for: Control of Sulfide Production in High Salinity Bakken Shale Oil Reservoirs by Halophilic Bacteria Reducing Nitrate to Nitrite
Source: Front Microbiol. 2017 Jun 21;8:1164. doi: 10.3389/fmicb.2017.01164 (PMC5478722; doi:10.3389/fmicb.2017.01164)
Supplement: Supplementary file 1 [file DataSheet1.docx]

Supplementary Material

**Control of sulfide production in high salinity Bakken shale oil reservoirs by halophilic bacteria reducing nitrate to nitrite**

Biwen Annie An, Yin Shen and Gerrit Voordouw*

*** Correspondence:** Gerrit Voordouw: Voordouw@ucalgary.ca

# Supplementary Figures and Tables

**Table S1** | **MPN for Bakken field sample.** The log MPN for source water (SW), injection water (IW), produced waters (PW), free-water knockout (FW) and treater waters (TW) from 2013 (11/13) and 2015 (01/15 or 08/15), at two separate salinities (0.01 and 2.0 M NaCl)


**Table S2 | Microbial community compositions for 11/13 samples from Bakken field.** The fraction (%) of pyrosequencing reads is given for the indicated taxa. The fractions in excess of 1% are in bold.

**Figure S1 | Linear correlation between salinity and ammonium for Bakken samples.**

**Figure S2 | Enrichment culture of nitrate-mediated souring control study using BS/4PW_11/13.** Three separate sets of experiment established to study nitrate-mediated souring control: sulfate with nitrate at 2.5 M NaCl, sulfate only at 2.5 M NaCl and sulfate with nitrate at 1 M NaCl. All experiments were established using 6 mM VFA, 10 mM sulfate and 10% inoculum of a primary enrichment of BS/4PW_11/13. Sulfate (A), sulfide (B), nitrate (C) and nitrite (D) are shown as a function of time.

**Figure S3 | Enrichment culture of nitrate-mediated souring control study using BS/9FW_11/13.** Three separate sets of experiment established to study nitrate-mediated souring control: sulfate with nitrate at 2.5 M NaCl, sulfate only at 2.5 M NaCl and sulfate with nitrate at 1 M NaCl. All experiments were established using 6 mM VFA, 10 mM sulfate and 10% inoculum of a primary enrichment of BS/9FW_11/13. Sulfate (A), sulfide (B), nitrate (C) and nitrite (D) are shown as a function of time.

**Figure S4 | Enrichment culture of nitrate-mediated souring control study using BS/10TW_11/13.** Three separate sets of experiment established to study nitrate-mediated souring control: sulfate with nitrate at 2.5 M NaCl, sulfate only at 2.5 M NaCl and sulfate with nitrate at 1 M NaCl. All experiments were established using 6 mM VFA, 10 mM sulfate and 10% inoculum of a primary enrichment of BS/10TW_11/13. Sulfate (A), sulfide (B), nitrate (C) and nitrite (D) are shown as a function of time.

**Figure S6 | Primary enrichments for BS_08/15 field samples.** The concentrations of sulfide (A) at 0.75 M and (B) 2.5 M NaCl with 10 mM sulfate & 20 mM lactate using 10 % inoculum

**Figure S5 | Primary enrichments for BS_01/15 field samples.** The concentrations of sulfide at 2.5 M NaCl with 10 mM sulfate & 20 mM lactate using 10 % inoculum

**Figure S7 | Primary enrichments for BS/2PW, 10TW, 13IW and 15PW_08/15.** Inoculation was set up with 20 mM nitrate and 6 mM VFA using 10 % inoculum at four separate salinities (0, 0.5, 1.5 or 2.5 M NaCl).

**Figure S8 | Reduction of sulfate and nitrate in chemostats.** Six chemostats were inoculated with lactate and sulfate at 2.5 M NaCl (A), VFA and sulfate at 2.5 M NaCl (B), lactate and sulfate at 0.5 M NaCl (C), lactate and nitrate and 2.5 M NaCl (D), VFA and nitrate at 2.5 M NaCl (E) or lactate and sulfate at 0.5 M NaCl (F).

**Figure S9 | Initial bioreactors using 2013 field samples.** (A) 6 mM VFA, 4 mM sulfate and 4 mM nitrate, (B) 6 mM VFA & 4 mM sulfate at 2.5 M NaCl
